# Incidences of Hypothyroidism Associated With Surgical Procedures for Thyroid Disorders: A Nationwide Population-Based Study

**DOI:** 10.3389/fphar.2019.01378

**Published:** 2019-12-12

**Authors:** Shin-Han Tsai, Shuo-Chen Chien, Phung-Anh Nguyen, Po-Han Chien, Hon-Ping Ma, Rahma Novita Asdary, Yao-Chin Wang, Ayesha Humayun, Chen-Ling Huang, Usman Iqbal, Wen-Shan Jian

**Affiliations:** ^1^Graduate Institute of Injury Prevention and Control, Taipei Medical University, Taipei, Taiwan; ^2^Emergency Department, Shuang-Ho Hospital, Taipei Medical University, Taipei, Taiwan; ^3^Graduate Institute of Biomedical Informatics, College of Medical Science and Technology, Taipei Medical University, Taipei, Taiwan; ^4^International Center for Health Information Technology (ICHIT), Taipei Medical University, Taipei, Taiwan; ^5^Department and Graduate Institute of Business Administration, National Taiwan University, Taipei, Taiwan; ^6^Master Program in Global Health and Development, PhD Program in Global Health and Health Security, College of Public Health, Taipei Medical University, Taipei, Taiwan; ^7^Department of Emergency, Min-Sheng General Hospital, Taoyuan, Taiwan; ^8^Department of Public Health and Community Medicine, Shaikh Khalifa Bin Zayed Al-Nahyan Medical College, Shaikh Zayed Medical Complex, Lahore, Pakistan; ^9^Division of Endocrinology and Metabolism, Department of Internal Medicine, Taipei Medical University Hospital, Taipei, Taiwan; ^10^School of Health Care Administration, Taipei Medical University, Taipei, Taiwan

**Keywords:** postoperative hypothyroidism, thyroid disorders, thyroid surgeries, transient hypothyroidism, surgeons experience, BigData analytics, hormones

## Abstract

**Background and Aim:** Limited information available about different types of thyroid surgeries with risk for postoperative hypothyroidism. This study aimed to investigate the risk of developing early and late-onset postoperative hypothyroidism in patients with thyroid disorders.

**Methods:** We used a large cohort data from the Taiwan National Health Insurance Research Data Base (NHIRDB) and identified 9,693 (9, 348) patients from January 1998 to December 2010, admitted for thyroid disorder surgeries. We used the surgical procedures time as the index date. Our observational retrospective cohort study excluded the subjects diagnosed with hypoparathyroidism and hypothyroidism before any surgeries. We analyzed the data using the Cox regression model to calculate the hazard ratio.

**Result:** Postoperative hypothyroidism associated with bilateral-total (HR, 4.27; 95% CI, 3.32–5.50), one-side total and another subtotal (HR, 3.16; 95% CI, 2.59–3.86), bilateral-subtotal (HR, 1.65; 95% CI, 1.37–1.98), and unilateral-total (HR, 1.17; 95% CI, 0.95–1.44) surgical procedures. The time intervals for thyroid disorders were 320 cases developed postoperative hypoparathyroidism in eight weeks, 480 cases the second month, and 1000 cases in the first year after surgery.

**Conclusion:** Findings suggest that thyroidectomy was associated with transient postoperative hypothyroidism in thyroid disorder patients. The bilateral-total surgical procedure was strongly associated with temporary postoperative hypothyroidism.

## Introduction

Thyroid dysfunction is common around the world. In endocrine practice, thyroid abnormalities take around 30% to 40% of the cases (Garmendia Madariaga et al., 2014). About 5% to 20% of American adult population has thyroid abnormalities (Bennedbæk and Hegedüs, 2000) with 1% to 2% in UK adults (Tunbridge et al., 1977) 17% to 35% in Brazilian women (Tomimori et al., 1995) 10% in Japanese adults (Kasagi et al., 2009) and 1 in 5788 live births occurring in Taiwan (Tsai et al., 1995).

Postoperative hypothyroidism is a major complication after thyroid disorders surgeries, appeared in 32.8% of the cases in the series reported by De Carlucci et al. (2008). Transient hypothyroidism incidence has been estimated to range from 6.9% to 46% (Falk et al., 1988; See and Soo, 1997; Mehrvarz et al., 2014) and permanent hypothyroidism from 0.4% to 33% (Thompson and Harness, 1970; Attie et al., 1979; Wingert et al., 1986; Falk et al., 1988) nevertheless, it depends on patients follow-up interval and their investigators in how they define hypothyroidism (Piper et al., 2005). Hypothyroidism constitutes of several complications such as basal calcification (Posen et al., 1979; Schafer and Ferbert, 1998), formation of cataract (Ireland et al., 1968) electrocardiographic abnormalities (Stathatos and Wartofsky, 2003) and tetany (Scanlon et al., 1981; Dembinski et al., 1994). Several studies reported that the transient and permanent postoperative hypothyroidism are associated with Graves’ disease (Van Welsum et al., 1974; FDA Drug Safety Communication, 2015; Sheehan and Doi, 2016), thyrotoxicosis (Querat et al., 2015) recurrent goiter (Wingert et al., 1986; Thomusch et al., 2000) and thyroid cancer (Pattou et al., 1998). Surgical techniques like devascularization or parathyroid glands inadvertent resection are associated with transient and permanent postoperative hypothyroidism (Elmaksoud. et al., 2015; Querat et al., 2015). The incidence of hypothyroidism related to different surgical procedures could be accomplished by estimating the risk of different surgical procedures. Despite of whether or not we know the behavior of patients after surgery their metabolism is still unpredictable.

Limited information exists about the relationship between different surgical procedures and risk to develop postoperative transient or permanent hypothyroidism which, for the most part is still unclear. Therefore, we aim to investigate different surgical procedures for thyroid disorders associated with transient or permanent hypothyroidism in the Taiwanese population.

## Materials and Methods

### Data Source

In this study, we used reimbursement data from the Bureau National Health Insurance (BNHI) system in Taiwan which was implemented on March, 1995 and has registered all the medical claims since 1996. More than 99% of Taiwan’s citizens are enrolled in the NHI, which offers mandatory and comprehensive medical care coverage to all Taiwanese residents (Hsing and Ioannidis, 2015). For research and administrative use, the National Research Institute established a randomly selected claim database which represents the whole population, and provides all information of medical services received by each individual yearly, from 1996 to 2012 (Lu and Hsiao, 2003). We randomly selected two million samples from Taiwan’s NHI beneficiary claim data during the years 1998 to 2011.

### Study Population

For our observational retrospective cohort study, we identified subjects from January 1, 1998 to December 31, 2010 who were hospitalized with surgeries for thyroid diseases [Taiwan National Health Insurance (NHI) codes 82001C, 82002C, 82004B, 82008B, 82015B, 82016B), and used the date of surgical procedures as the index date (see **Table S1** in Appendix). Moreover, subjects diagnosed with hypoparathyroidism and hypothyroidism before any surgical procedures, were excluded in this study. Initially, all eligible subjects were followed-up until a diagnosis of hypothyroidism [International Classification of Disease, Clinical Modification, Ninth Revision [ICD-9-CM) codes 244.0] or until the time subjects were censored for failure to follow-up, or termination of insurance, or a time beyond December 31, 2011 (see **Table S1** in Appendix).

### Covariate Assessment

The potential confounders were included in the study. The confounding factors influencing the risk of cancers such as age, gender, location (branch), and socio-economic status (SES) (based on the total amounts of payment to Taiwan’s National Health Insurance) were all included in this study. We also identified comorbidities that may be associated with mortality based on diagnostic codes from outpatient datasets prior to the outcome of interest. All diseases were included in the Charlson Comorbidity Index (CCI) and analyzed, except for human immunodeficiency virus (HIV) (Charlson et al., 1987).

### Data Analysis

One-way analysis of variance and independent t-test were used to compare each variable among groups undergoing surgery. A p-value of less than 0.05 was considered to be significant. Cumulative incidence curves were estimated by means of the method of Fine and Gray (Fine et al., 1999) were compared with the use of a log-rank test. Cox regression models with the duration (days) as the time scale were used to calculate hazard ratio (HR). The multivariable Cox model was adjusted for these confounders listed in **Table 1**. We used the SPSS 20 software to perform data analysis and the results calculations were expressed as the estimated numbers together with 95% confidence intervals (CIs).

**Table 1:** Characteristic of hypothyroidism patients for each surgical procedures.

	Subtotal		Total		One-side total and another- side subtotal	Radical with unilateral neck	p-value
	Unilateral	Bilateral	Unilateral	Bilateral			
**N**	1475	3037	2324	537	1216	306	–
**Gender, N (%)**							<0.001
**Female**	1192 (80.81)	2572 (84.69)	1857 (79.91)	441 (82.12)	981 (80.67)	240 (78.43)	
**Male**	283 (19.19)	465 (15.31)	467 (20.09)	96 (17.88)	235 (19.33)	66 (21.57)	
**Age**							<0.001
**Mean (SD)**	45.25 (13.87)	41.44 (14.17)	46.35 (14.04)	49.25 (13.49)	45.52 (14.4)	46.54 (14.3)	
**Comorbid conditions, N (%)**							
**Myocardial infarction**	11 (0.75)	18 (0.59)	24 (1.03)	4 (0.74)	5 (0.41)	3 (0.98)	0.337
**Congestive heart failure**	134 (9.08)	196 (6.45)	224 (9.64)	47 (8.75)	103 (8.47)	31 (10.13)	0.001
**Peripheral vascular disease**	56 (3.8)	98 (3.23)	106 (4.56)	34 (6.33)	65 (5.35)	15 (4.9)	0.002
**Cerebrovascular disease**	120 (8.14)	188 (6.19)	217 (9.34)	70 (13.04)	130 (10.69)	31 (10.13)	<0.001
**Dementia**	7 (0.47)	13 (0.43)	15 (0.65)	5 (0.93)	14 (1.15)	4 (1.31)	0.064
**COPD**	471 (31.93)	956 (31.48)	806 (34.68)	216 (40.22)	481 (39.56)	120 (39.22)	<0.001
**Rheumatic disease**	57 (3.86)	119 (3.92)	181 (7.79)	56 (10.43)	102 (8.39)	28 (9.15)	<0.001
**Peptic ulcer disease**	492 (33.36)	1042 (34.31)	897 (38.6)	251 (46.74)	540 (44.41)	132 (43.14)	<0.001
**Mild liver disease**	383 (25.97)	879 (28.94)	679 (29.22)	214 (39.85)	425 (34.95)	113 (36.93)	<0.001
**Diabetes**	172 (11.66)	326 (10.73)	354 (15.23)	120 (22.35)	211 (17.35)	54 (17.65)	<0.001
**Hemiplegia or paraplegia**	22 (1.49)	52 (1.71)	49 (2.11)	21 (3.91)	33 (2.71)	5 (1.63)	0.006
**Renal disease**	141 (9.56)	218 (7.18)	239 (10.28)	68 (12.66)	115 (9.46)	30 (9.8)	<0.001
**Cancer**	159 (10.78)	192 (6.32)	608 (26.16)	297 (55.31)	137 (11.27)	279 (91.18)	<0.001
**Moderate or severe liver disease**	0 (0)	2 (0.07)	5 (0.22)	3 (0.56)	1 (0.08)	0 (0)	0.023
**Charlson Comorbidities Index**							
**Mean (SD)**	3.09 (2.93)	2.63 (2.62)	3.79 (3.22)	5.28 (3.41)	3.63 (3.22)	5.53 (3.34)	
**Location, N (%)**							
**Taipei**	438 (29.69)	786 (25.88)	758 (32.62)	191 (35.57)	322 (26.48)	115 (37.58)	
**Northern**	138 (9.36)	334 (11)	330 (14.2)	52 (9.68)	123 (10.12)	24 (7.84)	
**Central**	238 (16.14)	1009 (33.22)	545 (23.45)	121 (22.53)	281 (23.11)	45 (14.71)	
**Southern**	257 (17.42)	477 (15.71)	310 (13.34)	60 (11.17)	173 (14.23)	59 (19.28)	
**Pingtung**	367 (24.88)	377 (12.41)	327 (14.07)	105 (19.55)	287 (23.6)	51 (16.67)	
**Eastern**	37 (2.51)	54 (1.78)	54 (2.32)	8 (1.49)	30 (2.47)	12 (3.92)	
**SES, N (%)**							<0.001
**INS_AMT< = 20,000**	686 (46.51)	1506 (49.59)	1044 (44.92)	220 (40.97)	563 (46.3)	129 (42.16)	
**20,000 < INS_AMT< = 40,000**	610 (41.36)	1231 (40.53)	890 (38.3)	217 (40.41)	491 (40.38)	130 (42.48)	
**INS_AMT > 40,000**	179 (12.14)	300 (9.88)	390 (16.78)	100 (18.62)	162 (13.32)	47 (15.36)	

### Ethical Approval

This type of study did not require the Institutional Review Board approval according to the policies of the National Health Research Institutes which provides large computerized de-identified data. http://nhird.nhri.org.tw/en/. This study contained unidentifiable living individual medical information, that the informed consent is not needed.

## Results

In this study, we included 8,895 patients who underwent thyroid diseases surgeries. The mean age of patients who undergoing unilateral-subtotal and bilateral-subtotal surgery were 45.25 and 41.25 years respectively. All the demographic characteristics, confounding comorbidities and other factors which could influence the outcomes of subjects are presented in **Table 1**. In our study analysis, we classified patients into six different thyroid disorder surgery groups and observed a maximum numbers of patients, where the bilateral-subtotal procedure group was compared to other groups. It is noticeable that among all surgery groups, female patients dominate all groups, ranging from 85% to 87%. We also observed the statistically significant difference in comorbid diseases, regional, and socioeconomic status (P < 0.001) among different surgical procedures groups.

**Figure 1:**
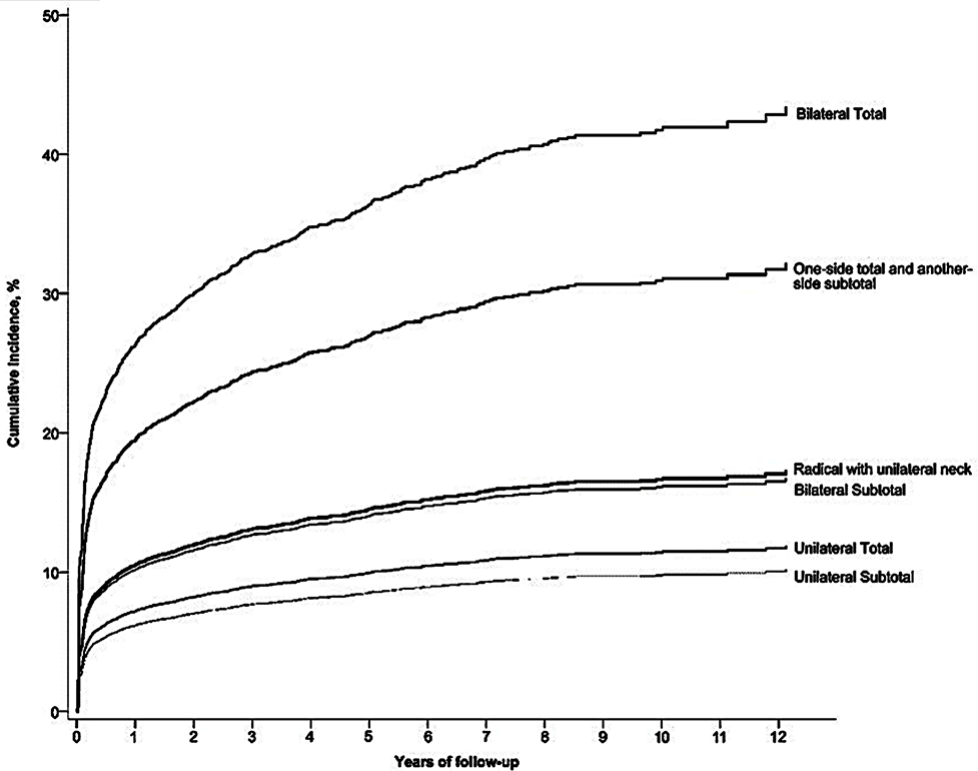
Cumulative incidences of hypothyroidism (ICD-9-CM code 244.0) from different surgical procedures.

We analyzed the cumulative incidence for hypothyroidism after adjusting confounding variables among different surgical procedures groups shown in **Figure 1**. It can be seen from the data in **Figure 1** that the bilateral total group revealed 45% cumulative incidence of postoperative hypothyroidism during the 12 years study period. There was no significant difference observed between the bilateral-subtotal (18%) and radical with unilateral neck (17.5%) surgery procedure group. Interestingly the unilateral-subtotal group presented the lowest rate of incidence (10%) among other surgical procedures groups for thyroid disorders.

**Figure 2:**
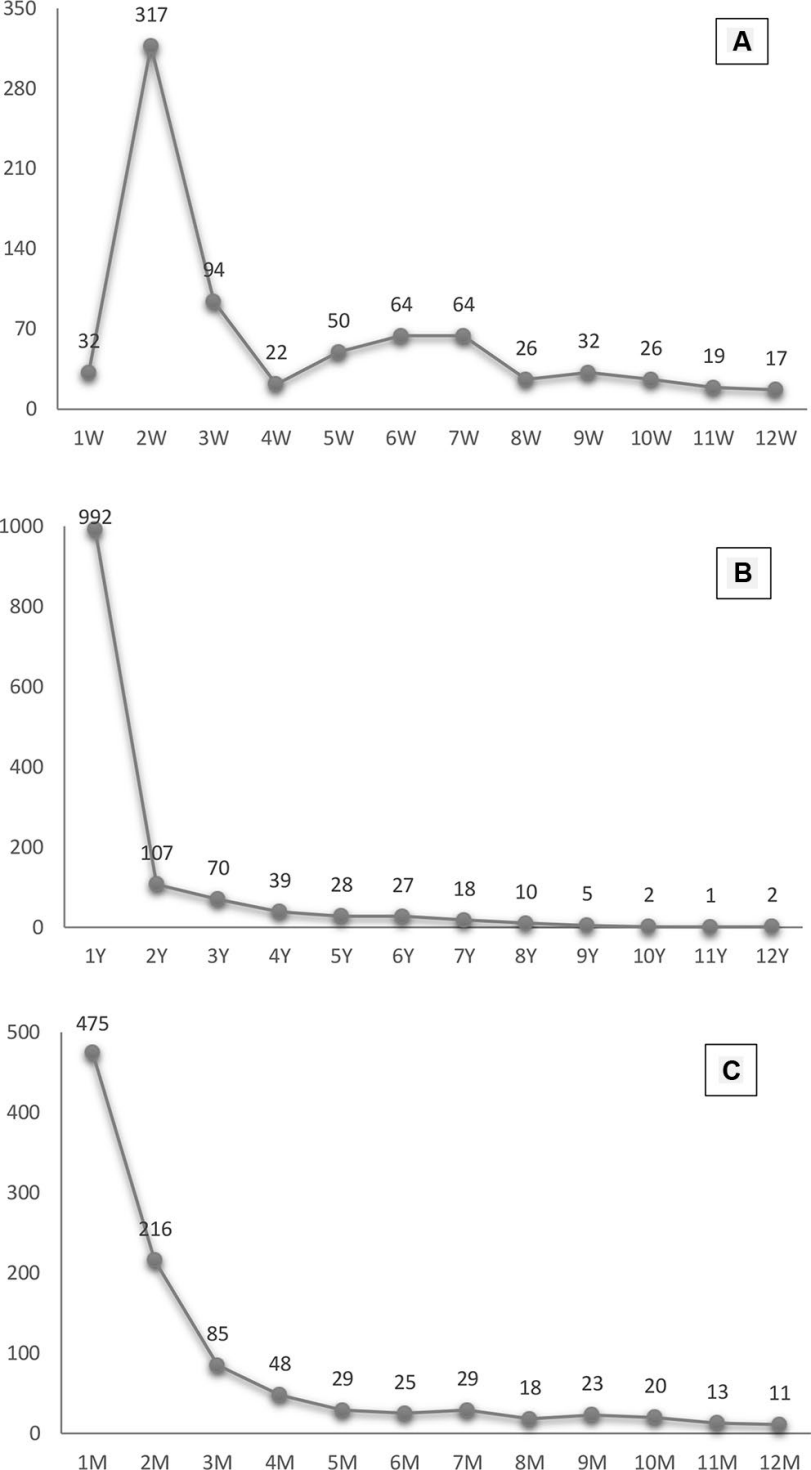
Postoperative hypothyroidism trends in patients for periods of weeks, months and years. **(A)** presents the incidence of hypothyroidism by week in first 12weeks. **(B)** presents the incidence of hypothyroidism by year. **(C)** presents the incidence of hypothyroidism by month.

**Table 2** presents incidence of developing hypothyroidism in all six surgical procedures groups. The significantly higher risk for developing hypothyroidism was observed in bilateral-total group (HR, 4.27; 95% CI, 3.32–5.50), for one-side total and another-side-subtotal (HR, 3.16; 95% CI, 2.59–3.86), and for bilateral-subtotal (HR, 1.65; 95% CI, 1.37–1.98) shown in **Table 2**. However, we did not observe any statistically significant association for unilateral-total (HR, 1.17; 95% CI, 0.95–1.44) with postoperative hypothyroidism. In this study, we also investigated the time interval trends associated with weeks, months and years for postoperative hypothyroidism. We found that among a total of 350 subjects after surgery for a period of 1–4 weeks, 340 developed hypoparathyroidism and which subsided within 8 weeks then patients were stable within 12 weeks (**Figure 2A**). Similar trends were observed for periods of 2–12 months while we followed 480 subjects post thyroid disorder surgery, in which symptoms subsided usually within the second month after surgery (**Figure 2B**). For longer periods (1–12 years), 1000 cases of post thyroid surgery became stable within the first year of surgery and no longer had symptoms by the ending of our study’s observation period. The rates of postoperative hypothyroidism were observed as significantly associated with increased occurrences just after surgical procedure but subsided shortly after, indicating a transient postoperative hypothyroidism as shown in **Figures 2A–C**. **Table 3** shows the relation of thyroid disorders and the surgical procedures which surgeon selected to perform.

**Table 2:** Hypothyroidism risk after adjusting for surgical procedures.

Operation procedures	No.	Hypothyroidism No.	HR (95% CI)*	P-Value
Unilateral Subtotal	1475	153	148	
Bilateral Subtotal	3037	484	1.65 (1.37 - 1.98)	<0.001
Unilateral Total	2324	219	1.17 (0.95 - 1.44)	0.145
Bilateral Total	537	126	4.27 (3.32 - 5.50)	<0.001
One-side total and another-side subtotal	1216	289	3.16 (2.59 - 3.86)	<0.001
Radical with unilateral neck	306	31	1.70 (1.13 - 2.57)	0.011

*Adjusted for variables as in **Table 1**.

**Table 3:** The relation of thyroid disorders and the surgical procedures which surgeon selected to perform (Hypothyroidism).

Surgical Procedure/Thyroid disorders (ICD-9 code)	Unilateral Subtotal N = 1475	Bilateral Subtotal N = 3037	Unilateral Total N = 2324	Bilateral -Total N = 537	One-side total and another- side subtotal N = 1216	Radical with unilateral neck N = 306	Total
193 (Malignant neoplasm)	63 (4.06%)	75 (2.33%)	419 (17.38%)	265 (45.06%)	57 (4.55%)	275 (88.14%)	1154
240 (Simple and unspecified goiter)	1 (0.06%)	0	0	0	0	0	1
240.0 (Goiter, specified as simple)	4 (0.26%)	4 (0.124%)	4 (0.16%)	0	0	0	12
240.9 (Goiter, unspecified)	74 (4.18%)	82 (2.54%)	93 (3.85%)	8 (1.36%)	53 (4.23%)	1 (0.32%)	311
241 (Nontoxic nodular goiter)	4 (0.26%)	3 (0.09%)	0	0	0	0	7
241.0 (Nontoxic uninodular goiter)	137 (8.91%)	44 (1.36%)	225 (9.33%)	3 (0.51%)	31 (2.47%)	11 (3.52%)	451
241.1 (Nontoxic multinodular goiter)	133 (8.65)	1048 (32.56%)	246 (10.20%)	141 (23.97%)	340 (27.15%)	2 (0.64%)	1910
241.9 (Unspecified nontoxic nodular goiter)	654 (42.57%)	504 (15.66%)	867 (35.97%)	53 (9.01%)	278 (22.20%)	4 (1.28%)	2360
242 (Thyrotoxicosis with or without goiter)	0	5 (0.15%)	0	0	0	0	5
242.0 (Toxic diffuse goiter)	0	11 (0.34%)	1 (0.041%)	0	0	0	12
242.1 (Toxic uninodular goiter)	0	0	0	0	0	0	0
242.2 (Toxic multinodular goiter)	0	0	0	0	0	0	0
242.3 (Toxic nodular goiter unspecified type)	1 (0.06%)	0	0	0	0	0	1
242.4	0	0	0	0	0	0	0
242.8 (Thyrotoxicosis of other specified origin)	0	0	0	0	0	0	0
242.9 (Thyrotoxicosis without mention of goiter or other cause)	0	1 (0.03%)	0	0	0	0	1
Other diseases	404 (27.39%)	1260 (41.49%)	469 (20.18%)	67 (12.48%)	457 (37.58%)	13 (4.25%)	2670

## Discussion

We investigated postoperative hypothyroidism’s association with thyroid disorder surgeries in the Taiwanese population. The patients with thyroid disorders undergoing six different surgical procedures allowed us to understand the incidence of postsurgical hypothyroidism among thyroidectomies. To our best knowledge, this study provided evidence of association using a large cohort population sample with different surgical procedures for thyroid disorders. Patients’ proportion who developed postoperative hypothyroidism is substantial and varying from 10% to 45% depending on the type of surgical procedure.

The findings were startling in that 23.46% were bilateral total 23.76% one-side total and another-side-subtotal surgery associated with greater risk to postoperative hypothyroidism as compared with unilateral-subtotal. However, no significant association was observed in patients with a unilateral total surgical procedure for hypothyroidism. These findings show that the more extensive and substantial surgical procedure would have greater risk to develop postoperative hypothyroidism. Nevertheless, all patients encountered postoperative hypothyroidism temporarily which subsided during the study period which indicates a transient hypothyroidism. Our findings are consistent with Rosato et al. (2004), Michie et al. (1972) and Dunn and Chapman, (1964) that hypothyroidism was a temporary condition and did not reoccur after more than one year post thyroidectomy. However, some studies reported that most of the subtotal thyroidectomy patients showed hypothyroidism after surgery as the long-term outcome (Sung et al., 2015). Hedley et al. (1983) reported that patients undergoing subtotal thyroidectomy are not protected against early or late postoperative hypothyroidism.

In this study, 1048 patients with non-toxic multinodular goiter underwent bilateral-subtotal surgery, 867 patients with unspecified nontoxic nodular goiter had unilateral total surgery and 867 patients underwent thyroidectomy among other thyroid disorders (see **Table 3**). It is revealed that among surgeries, bilateral-subtotal surgical procedures were the most common we observed, with 32.56% patients having non-toxic multinodular goiter. However, most of the patients with thyroid cancer received total thyroidectomy and radioiodine ablation therapy. The reason for these procedures is to prevent a thyroid disorder from becoming hypothyroidism. Usually, multinodular goiter is less likely to develop into thyroid cancer but unilateral nodular goiter has a relatively higher thyroid cancer incidence as compared to multinodular. This might be one of the important reasons that these patients receive unilateral surgery more frequently.

Johner et al. (2011) reported that the incidence of hypothyroidism following thyroid lobectomy is low, and a significant proportion of individuals who become biochemically hypothyroid will reveal only a transient elevation in their TSH levels. We observed that radical thyroidectomy with unilateral neck lymph node dissection had increased incidence of patients with thyroid cancer (88.14%) compared to bilateral total surgical procedure (45.06%). All types of thyroid cancer such as papillary, follicular, medullary, or anaplastic could be removed by using complete thyroid resection surgery. In some cases, if the tissues could not be fully removed, then radioactive iodine therapy is often used to destroy the tissues.

In this current study, we also investigated cumulative incidences of post-operative hypothyroidism in patients undergoing surgery for any thyroid disorder during a time period of weeks, months and years. For a time period of 1–4 weeks, 340 patients developed postoperative hypothyroidism during the second week following thyroid related surgery which subsided to normal within 3 to 8 weeks. For a period of 2–12 months, 480 patients developed postoperative hypothyroidism and which subsided in 2–3 months after thyroid surgery. Similar trends were observed for periods of 1–12 years where almost 1000 patients underwent surgery and developed postoperative hypothyroidism only shortly after surgery, which subsided within the 2–12 years of our study period. Interestingly, we observed almost the same trends for different periods (weeks, months and years) which showed a transient hypothyroidism after surgery for temporarily which subsided afterwards. Our findings are consistent with previous studies in that hypothyroidism is associated with total thyroidectomy and occurred frequently, however, it could be managed as compared to hypoparathyroidism (Mortimore et al., 1998). Verloop et al.’s meta-analysis (Yang et al., 2010) showed that approximately one in five patients will develop hypothyroidism after hemithyroidectomy, with clinical hypothyroidism in one of 25 patients undergoing surgery. Tomoda et al. (2011) observed a 70% incidence of hypothyroidism associated with hemithyroidectomy.

Thomusch et al. (2003) reported that the surgical techniques and extent of resection had a greater influence on permanent postoperative hypoparathyroidism than thyroid pathologic condition. Oda et al. (2016) found that 16.7% transient hypoparathyroidism is associated with tumor enlargement and the appearance of novel lymph node metastases surgeries. Okamoto et al. (1992) and Sugino et al. (1993) reported that 10% of the patients encountered an unexpected permanent postoperative hypothyroidism, despite choosing a surgical procedure to avoid drug usage for longer periods. While medical therapy with an anti-thyroid drug is commonly adopted in European nations and Japan as the first-choice method of therapy, the disease still often occurs. Recent systematic review reported the clinical, behavioral and pharmacogenomic factors could be influence in response to levothyroxine therapy in patients with primary hypothyroidism (Dew et al., 2017).

Moreover, the literature on the opinions of thyroid surgeries experts is somewhat controversial. Sosa et al. (1998) and Shindo et al. (1995) reported that surgeons experience is associated with complication rates. We observed in thyroid cancer patients that half of them took conservative surgeries (unilateral-subtotal, bilateral-subtotal and unilateral-total) and more than half took aggressive surgery (bilateral-total, unilateral-total and unilateral-subtotal, and radical thyroidectomy) with unilateral neck lymph node. The surgical procedure often took place in consideration to the cancer cell type, and stage and size of nodules. After thyroidectomy, the transient hypothyroidism often occurring in patients is associated with blood loss during surgery. This usually subsides once the tissue regeneration and blood perfusion occurs, which leads to the thyroid gland functions returning to normal.

The findings of this study should be interpreted by acknowledging that we did not have access to the type of cancer cells, tumor stage and nodules size information, as we used Taiwan NHI database which only contains claims data. Some limitations may be inevitable in this retrospective NHIRD study. However, our study was still valuable because it is a population-based nationwide long-term study and the NHIRD records included a large sample size (1 million random individuals) with the general representation (covered more than 99% Taiwan citizens). Moreover, the National Health Insurance is a nationwide legislative policy based on National Health Insurance Act and is governed by National Health Insurance Administration, Ministry of Health and Welfare, Republic of China (Taiwan). Insurance claims are scrutinized by official medical specialists and monitored by peer reviewers according to standard diagnostic criteria. Although we used multiple methods during the inclusion process to identify the diagnosis and to minimize misclassification, a few atypical cases may still present difficulties in classification and this issue we further try to deal by ensuring the patients long term follow-up.

## Conclusion

The most significant findings to emerge from this study is that thyroidectomy was associated with transient postoperative hypothyroidism in thyroid disorder patients. The research has also shown that the risk was associated with different surgical procedures for thyroid disorders as well. Postoperative hypothyroidism usually occurs only temporarily and subsides afterwards.

## Data Availability Statement

The raw data supporting this manuscript’s findings will be available upon request to any qualified scientist by the authors, without undue reservation.

## Author Contributions

W-SJ designed the study, enrolled patients, interpreted data, wrote the report, and approved the final draft. UI designed the study, searched the published work, analyzed and interpreted data, reviewed the manuscript, and approved the final draft. H-PM, Y-CW and P-HC recruited patients, collected and interpreted data, reviewed the manuscript, and approved the final draft. P-AN, C-LH, AH, W-SJ and UI interpreted data, reviewed the manuscript, and approved the final draft. C-LH, S-CC and RNA recruited participants, reviewed the report and approved the final draft. S-HT designed the study, analyzed and interpreted data, reviewed the report, and approved the final draft.

## Funding

This research was supported by Taipei Medical University – Shuang-Ho Hospital, Ministry of Health and Welfare Project number 105TMU-SHH-11, YUAN’s Hospital project number 107YGH-TMU-10, and Ministry of Science of Technology project number MOST107-2218-E-038-004-MY2.

## Conflict of Interest

The authors declare that the research was conducted in the absence of any commercial or financial relationships that could be construed as a potential conflict of interest.
